# Prevalence of erosive lesions with respect to risk factors in a young adult population in Poland—a cross-sectional study

**DOI:** 10.1007/s00784-016-2012-z

**Published:** 2016-12-15

**Authors:** Izabela Strużycka, Adrian Lussi, Agnieszka Bogusławska-Kapała, Ewa Rusyan

**Affiliations:** 1 Department of Comprehensive Dental Care, Warsaw Medical University, ul. Miodowa 18, 00-246 Warsaw, Poland; 2 Department of Preventive, Restorative and Pediatric Dentistry, University of Bern, Freiburgstrasse 7, CH-3010 Bern, Switzerland; 3 Department of Conservative Dentistry, Warsaw Medical University, ul. Miodowa 18, 00-246 Warsaw, Poland

**Keywords:** Dental erosion, Epidemiology study, Risk factors, BEWE

## Abstract

**Objective:**

The study was conducted to investigate the prevalence of erosive lesions and related risk factors in the population of 18-year-old young adults in Poland.

**Materials and methods:**

Calibrated examiners measured erosive tooth wear according to Basic Erosive Wear Examination (BEWE) scoring system in 1869 patients and assessed the impact of risk factors with a questionnaire.

**Results:**

Erosion was present in 42.3% of the patients. Early damage to the enamel was the most frequent finding (BEWE 1)—28.9%. More advanced lesions (BEWE 2) were observed in 12% of the patients. Advanced damage to the teeth (BEWE 3) was diagnosed rarely—1.4% of the examined population. Acidic diet, hygienic habits, and medical conditions such as asthma, eating disorders, and esophageal reflux showed statistical significance, as associated with erosion in the examined population.

**Conclusions:**

The present study indicates that dental erosion is a common oral disease in the 18-year-old population with prevalence of frequency and intensity in males. However, on the basis of observations carried out in recent years, it may be assumed that the prevalence of this type of lesion is increasing.

**Clinical relevance:**

Present findings support other longitudinal studies toward evaluation of the BEWE system as a valuable standard for assessing erosive and related risk factors among different populations.

## Introduction

Dental erosion is a process characterized by the irreversible loss of hard dental tissue due to chronic and localized action of acids and chelating agents [1]. Endogenous and exogenous factors are recognized as contributing to this process. The former include presence of gastric acid, composition and amount of secreted saliva, composition of acquired dental pellicle, anatomy of teeth and adjacent tissues, and lastly quality of general health. The latter are diet, lifestyle, hygiene practices/habits, physical activity, impact of the environment, and one’s occupation [2–5]. The progression of lesions is a result of the resistance of hard dental tissues to all of the above-mentioned factors and the duration of their exposure to dental surfaces.

The increased morbidity that has been observed in recent years may be an indication that dental erosion has become an oral health problem that cannot be ignored in industrialized and developing countries. European epidemiological studies clearly indicate that the issue concerns all age groups; however, the most spectacular rise in prevalence has been observed in teenage and young adult populations, which is probably due to a change in eating habits and lifestyle [6–9].

In Poland, a nationwide survey on the prevalence of noncarious lesions was conducted for the first time in 2011. The study examined a population of 15-year-old adolescents, in which the presence of this type of defect was observed to a varying degree in 25% of the examined subjects. Early enamel lesions were observed predominantly, while more profound lesions were rare and did not exceed 3% of the population [10]. The obtained results of the epidemiological studies prompted further examinations, this time on a population of 18-year-old adolescents. At the same time, apart from evaluating the prevalence and extent of erosive lesions, this study attempted to define sociodemographic factors and hygienic and dietary habits as potentially contributing to the progression of the disorder.

The present study aimed at assessing the prevalence of dental erosion and associated risk factors in a population of 18-year-old young adults in Poland.

## Materials and methods

The clinical study investigating the prevalence of erosion in the aforementioned population was carried out within the framework of the Nationwide Monitoring of Oral Heath in Poland, which was implemented for the first time in Poland in 1997. These epidemiological surveys are performed according to the Oral Health Surveys criteria by WHO.

The program was run in cooperation with the Ministry of Health and the Medical University of Warsaw and provided an assessment of the current health situation of the Polish population as well as defining changes that were affecting health and its determinants. Since then, a periodic epidemiological survey has been performed on specific age groups, groups with high risk of oral diseases, or beneficiaries of specific prophylactic programs.

The sample was chosen by means of a step-wise sampling, beginning with a random choice of seven out of 16 Polish provinces, followed by stratified random sampling of one rural and one urban county in each of these provinces. The next step of the sampling procedure included a random choice of two secondary schools in each county. Finally, two classes were randomly selected in each of the schools. Then, within each class, all pupils present on the days of the survey and fulfilling the age criteria were designated for examination. In each region, the examinations were conducted by two dentists and all examiners were initially trained and calibrated. The level of agreement of at least 85% (Kappa Statistics) was obtained for the registration of dental erosion in all surveys. The multilayer, stratified random sampling process and the height rates of participation allowed the authors to assume that the selected sample was representative of the young Polish population. A total of 1869 subjects participated in the study, which included 947 females and 922 males (55.3% came from an urban background). Clinical assessment of the dentition used criteria based on the Basic Erosive Wear Examination (BEWE) index. The occurrence of noncarious erosive lesions was diagnosed on labial/buccal, lingual/palatal, and occlusal surfaces of all the teeth present in the mouth, excluding the third molars. The examination, naturally, omitted the teeth that were missing, restored (more than 50% of the tooth surface), damaged, or destroyed by caries. Subjects wearing braces were also excluded. Visual examination of the mouth and teeth was carried out in sextants according to the following divisions: 17–14, 13–23, 24–27, 37–34, 33–43, and 44–47. The BEWE index categorizes the progression of erosive lesions into four grades: 0 (no lesion), 1 (slight enamel damage), 2 (damage affecting less than 50% of tooth surface), and 3 (loss of more than 50% of the tooth surface in the examined sextant). The teeth were examined in dental surgeries in artificial light.

After the examination, each subject filled out a specially prepared questionnaire with 40 items that covered general medical problems such as reflux, eating disorders, asthma, allergies, medication, oral hygiene, and dietary habits.

The data were analyzed with descriptive, bivariate, and multivariate methods. Chi-square test was used for comparison of proportions and Spearman’s coefficient was used to analyze correlations. The multivariate analysis was performed using the multivariate step-back regression method. The independent variables were gender, residential environment, gastroesophageal reflux, allergies, asthma, toothbrush type, and tooth brushing time and frequency, as well as consumption of dietary acids. The dependent variables were maximum erosive tooth wear and localization of erosive tooth wear (anterior/posterior teeth). The level of significance was set at *p* = 0.05 for bivariate analyses and for models generated by means of multivariate analyses, while for individual variables included in these models it was set at *p* = 0.2.

Ethical approval was obtained from the Medical University of Warsaw Bioethics Committee.

## Results

On the basis of clinical examinations, it was found that the percentage of healthy subjects in the analyzed population was 57.7%. Erosion was present in 42.3% of the patients. Early damage to the enamel (BEWE 1) was the most frequent finding at 28.9%. More advanced lesions (BEWE 2) were observed in 12% of the patients. Advanced damage to teeth (BEWE 3) was diagnosed rarely and did not exceed 1.4% of the examined population of young adults. Lesions occurred in 421 out of 922 males, i.e., in 45.7% of the males. Accordingly, affected were 370 out of 947 women, i.e., 39.0% of the females, and the difference was statistically significant. This trend was particularly apparent with respect to more profound lesions (BEWE 2 and 3). The percentage of the male subjects whose lesions were more profound was 16.6% compared to 10.4% of the females. Additionally, the place of residence affected the clinical characteristics of the examined population. A lower percentage of patients with erosion was observed in rural areas compared to urban areas; the differences, however, were not statistically significant. Values obtained for the BEWE index are presented in Table 1. In the examined population, erosive lesions were more often localized in the anterior region compared to lateral segments of the dental arches (35.3 and 19.8%, respectively). Yet, even though lesions were less frequent laterally, they were more prominent. Also, subjects with advanced lesions in the anterior teeth demonstrated increased progression of erosive lesions in lateral segments. This correlation was statistically significant, but only slightly (*r* = 0.54) (Table 2).

**Table 1.** Low and high values of the BEWE index in 18-year-old subjects

	BEWE = 0–1	BEWE = 2–3	Comparison (chi-square)
Males (*n* = 922)	769 83.4	153 16.6	*p* < 0.001
Females (*n* = 947)	849 89.6	98 10.4	
City (*n* = 1033)	890 86.2	143 13.8	*p* = 0.56
Village (*n* = 836)	728 87.1	108 12.9	
Total (*n* = 1869)	1618 86.6	251 13.4	–

**Table 2.** Comparison of the presence of erosion in the anterior and posterior segments of the dental arch (BEWE index median for the anterior and posterior sextants in the maxilla and the mandible)

Median BEWE	0	0.5–1	1.5–2	2.5–3	Comparison (chi-square)
Anterior segment	1209 64.7%	492 26.3%	156 8.3%	12 0.6%	*p* < 0.0001
Posterior segment	1498 80.2%	281 15.0%	71 3.8%	19 1.0%	

Every subject who participated in the clinical examination was asked to complete a questionnaire on potential risk factors for dental erosion. When asked to subjectively assess their own general health, most respondents marked the option “good.” For the question “Are you generally healthy?” there were 85.7% positive answers and 11.6% negative ones. Allergies and asthma were the most frequently reported conditions at 8.5 and 2.7%, respectively. A relatively small percentage of patients reported gastroesophageal reflux and eating disorders (1.3 and 1.4%, respectively). Statistical analysis revealed that the prevalence of erosion in the anterior region was related to gastroesophageal reflux, eating disorders, and asthma at the significance level of *p* < 0.05. In addition, the presence of lesions in the lateral segments was correlated with patients who reported reflux (*p* < 0.05). Relevant data are presented in Table 3.

**Table 3.** Occurrence of erosion in the anterior and posterior segment of the dental arch related to systemic diseases as reported by respondents

Do you suffer from:	Dental segment		Median BEWE				Comparison (chi-square)
			0	0.5–1	1.5–2	2.5–3	
Gastroeso-phageal reflux?	Anterior	Yes	15 62.5%	5 20.8%	3 12.5%	1 4.2%	*p* < 0.05
		No	1194 64.7%	487 26.4%	153 8.3%	11 0.6%	
	Posterior	Yes	19 79.2%	4 16.7%	0 0%	1 4.2%	*p* < 0.05
		No	1479 80.2%	277 15.0%	71 3.8%	18 1.0%	
Eating disorder?	Anterior	Yes	17 58.6%	8 27.6%	3 10.3%	1 3.4%	*p* < 0.05
		No	1192 64.8%	484 26.3%	153 8.3%	11 0.6%	
	Posterior	Yes	23 79.3%	4 13.8%	1 3.4%	1 3.4%	*p* = 0.08
		No	1475 80.2%	277 15.1%	70 3.8%	18 1.0%	
Allergy?	Anterior	Yes	120 65.2%	44 23.9%	17 9.2%	3 1.6%	*p* = 0.06
		No	1089 64.6%	448 26.6%	139 8.2%	9 0.5%	
	Posterior	Yes	143 77.7%	27 14.7%	9 4.9%	5 2.7%	*p* = 0.13
		No	1355 80.4%	254 15.1%	62 3.7%	14 0.8%	
Asthma	Anterior	Yes	31 56.4%	17 30.9%	5 9.1%	2 3.6%	*p* < 0.01
		No	1178 64.9%	475 26.2%	151 8.3%	10 0.6%	
	Posterior	Yes	42 76.4%	9 16.3%	2 3.6%	2 3.6%	*p* = 0.19

Subsequent questions aimed at determining the dietary preferences of the examined individuals as well as the frequency of consumption of acidic foodstuffs. These primarily included those with well-documented erosive potential such as fruit and fruit juices, carbonated beverages, and fruit teas. Statistical analysis of provided answers revealed that frequent consumption of acidic foodstuffs, especially fruit teas and energizing beverages, favored the development of erosion in the anterior segment of the dentition. Laterally, erosion was more frequently confirmed in individuals drinking fruit teas and isotonic beverages. Analysis of median values clearly demonstrated that acidic dishes and beverages in the examined population constituted a strong risk factor for progression and intensification of erosion in the entire dentition at the level of statistical significance (Table 4).

**Table 4.** Correlation between the presence of erosion and consumption of acidic foodstuff (Spearman’s correlation analysis)

	BEWE max	Number of sextants with erosion	Erosion in the anterior segment	Erosion in the posterior segment
Fruit	*p* = 0.66	*p* = 0.49	*p* = 0.19	*p* = 0.30
Fruit juices	*p* = 0.80	*p* = 0.81	*p* = 0.80	*p* = 0.98
Fruit teas	*r* = 0.06 *p* < 0.05	*r* = 0.01 *p* < 0.05	*r* = 0.05 *p* < 0.05	*r* = 0.05 *p* < 0.05
Isotonic drinks	*p* = 0.15	*p* = 0.09	*p* = 0.28	*r* = 0.06 *p* < 0.01
Carbonated beverages	*r* = 0.08 *p* < 0.001	*r* = 0.08 *p* < 0.001	*r* = 0.08 *p* < 0.001	*r* = 0.05 *p* = 0.05
Energizing drinks	*p* = 0.93	*p* = 0.92	*p* = 0.61	*p* = 0.75
Pickles	*p* = 0.63	*p* = 0.91	*p* = 0.90	*p* = 0.28
Acidic solids and liquids—median values	*p* = 0.08	*r* = 0.04 *p* < 0.05	*r* = 0.04 *p* < 0.05	*p* = 0.70

As for responses concerning hygienic practices, their analysis revealed that the presence of anterior lesions was related to both hardness of toothbrush fibers and its type (manual vs electric) (Table 5). However, no correlation was observed between the presence of erosive lesions and time that elapsed from the moment a meal was finished.

**Table 5.** Relationship between the type of toothbrush used by a respondent and the presence of erosion in the anterior segment of dental arch

Toothbrush used	Median BEWE				
	0 (%)	0.5–1 (%)	1.5–2 (%)	2.5–3 (%)	Comparison (chi-square)
I don’t use any	42.9	42.8	14.3	0.0	*p* < 0.05
Manual hard	61.0	27.1	11.9	0.0	
Manual medium	66.8	25.4	6.9	1.0	
Manual soft	65.6	25.7	8.3	0.4	
Electric	60.45	27.8	11.8	0.0	

Multivariate analysis revealed that erosion in the anterior teeth was statistically significantly associated with gender and place of residence, while in the posterior teeth, as well as in case of maximum BEWE value, the only independent factor was gender (Tables 6 and 7). It should be noted though, that the consumption of acidic solids and liquids almost reached statistical significance (*p* = 0.059), suggesting it can also play a substantial role, especially in the anterior tooth segment.

**Table 6.** Multivariate analysis: model of risk factors for the occurrence of dental erosion (dependent variable max BEWE = 2–3 vs max BEWE = 0–1)

Independent variable	Standardized beta coefficient	Significance level	OR
Gender (males vs females)	0.08	*p* = 0.001	OR = 1.71 (95% CI: 1.30–2.27)
Consumption of acidic solids and liquids (at least four types of acidic foodstuff consumed at least once daily)	0.05	*p* = 0.059	OR = 1.08 (95% CI: 0.79–1.48)
Asthma	0.03	*p* = 0.137	OR = 1.34 (95% CI: 0.66–2.71)
Place of residence (city vs village)	0.03	*p* = 0.147	OR = 1.03 (95% CI: 0.79–1.48)

R^2^ = 0.01, *p* < 0.0001

**Table 7.** Multivariate analysis: model of risk factors for the occurrence of dental erosion (dependent variable BEWE = 2–3 within the anterior or posterior teeth)

Anterior segment			
Independent variable	Standardized beta coefficient	Significance level	OR
Gender (males vs females)	0.07	*p* = 0.004	OR = 1.68 (95% CI: 1.20–2.34)
City vs village	0.05	*p* = 0.048	OR = 1.06 (95% CI: 0.77–1.47)
Consumption of acidic solids and liquids (at least four types of acidic foodstuff consumed at least once daily)	0.04	*p* = 0.083	OR = 1.34 (95% CI: 0.94–1.91)
Asthma	0.04	*p* = 0.125	OR = 1.38 (95% CI: 0.61–3.13)
Posterior segment			
Independent variable	Standardized beta coefficient	Significance level	OR
Gender (males vs females)	0.08	*p* = 0.0006	OR = 2.02 (95% CI: 1.30–3.16)
Allergy	0.03	*p* = 0.142	OR = 1.75 (95% CI: 0.97–3.17)

## Discussion

The 40% prevalence of erosive lesions evaluated on the basis of the Nationwide Monitoring of Oral Heath in Poland examinations was much higher than that previously described in the literature. Studies conducted at the turn of the twenty-first century showed that the percentage of young adults with damage to hard dental tissues was in the range of 2.38 to 15% [11, 12]. Also, studies in 2011 on 15-year-old adolescents revealed an increased prevalence and intensity of dental erosion [10]. Similar trends have been observed in many European countries and the USA. The results clearly indicate that erosion, especially in teenage populations, is on the rise [8, 13–15]. Despite the fact that Polish results are still below those in highly developed countries, they still reflect a continued worsening of oral health in this age group compared to previous evaluations. Bearing in mind that the occurrence of noncarious lesions increases with age, it can be assumed that the functionality and esthetics of the dentition in this population may deteriorate with time.

In spite of the prevalence of dental erosion in 18-year-old adolescents in Poland is slightly below the prevalence for other nationalities, the interpretation of the obtained results poses difficulties due to differences in applied protocols, calibration of the examiners, the use of different indices, and the lack of uniformity of the examined populations [16]. This is the reason why the standardized examinations carried out in seven European countries on a sample of 3187 subjects aged 18–35 years by means of the BEWE index are so valuable. It was observed that the percentage of subjects with the highest BEWE values (2 and 3) ranged from 17.7 to 54.4%. The higher percentage of the patients with erosive lesions compared with that of the present study may be attributed to a wider age range of the sample on the one hand and significant differences in individual participating countries on the other. It is also worth noting that former communist countries such as Lithuania and Latvia obtained results similar to those of the Polish ones [13]. In the Polish sample, erosion prevailed in males and this difference was particularly evident for more profound lesions. A higher prevalence of noncarious lesions in males has also been suggested by many other authors [17, 18]. The observed differences may have been due to other sociodemographic and cultural characteristics of patients and to different research methodologies.

Statistical analysis of responses to questions revealed that established systemic risk factors such as gastroesophageal reflux, eating disorders, and asthma were significantly associated with the development of noncarious lesions, as reported by other authors [19–23]. The correlation of gastroesophageal reflux and erosion has been discussed at length for many years, yet it has not been conclusively resolved. Meta-analysis based on scientific publications revealed that people suffering from gastroesophageal reflux disease have an increased risk of dental hard tissue damage due to the action of gastric acids, which has also been documented in the present study [24].

The questionnaire did not include a question directly inquiring about regurgitation and vomiting since it had been revealed by previous pilot studies that approximately 60% of the respondents fail to respond to that question. It is still hard to elicit reliable answers to questions about these disorders. Epidemiological studies have demonstrated that the prevalence of eating disorders in Poland is comparable to that in Western Europe, and the subclinical type accounts for 2.34% of the population and for 28.6% in individuals who maintain a strict dietary regimen [25]. In the present sample, less than 1.4% of the respondents suffered from eating disorders, and almost half of that number manifested erosion-type damage to teeth of varying intensity, generally corroborating other studies [26, 27].

Reports on the risk of dental erosion in asthmatics are conflicting. There are several authors whose observations do not confirm such a correlation [28, 29]. The present study found a higher prevalence of erosion in that group of patients at a level of statistical significance. Likewise, Jain et al. reported an increased prevalence of lesions in asthmatics, which they attributed to a side effect of administered medication, namely reduced pH in the mouth [30].

A diet rich in acidic foodstuffs and beverages is a well-documented risk factor for erosive damage to the teeth. An analysis of the specific foodstuffs that were listed in the questionnaire showed that only fruit teas and carbonated beverages were associated with the occurrence of dental erosion. It is likely that due to the geographic location and the seasonal availability of specific foodstuffs, no significant correlation was found linking fruit and fresh fruit juices with tissue damage. At the same time, a collective analysis of dietary preferences of the examined population clearly demonstrated that both the type of food and the frequency of consumption had a significant impact on the emergence of dental erosion, corroborating other studies [5, 31, 32]. It is, therefore, of utmost importance to analyze patients’ diet in detail, in particular in cases of noncarious lesions, so that prophylactic and therapeutic measures can be adjusted to the local population and undertaken to inhibit the progression of this condition.

Epidemiological studies carried out in seven European countries showed that the patients’ hygiene practices had no significant impact on tooth damage formation as previously thought [13]. This is due, on one hand, to the protective role of toothpaste, which is rich in remineralizing compounds, and, on the other hand, to increasing health awareness among patients [33]. It has to be noted that inaccurate instruction concerning the duration of brushing may adversely affect other health-promoting activities performed by patients and, for example, exacerbate dental caries. The present study indicates that the time period between a meal and tooth brushing did not influence the prevalence or severity of lesions in the examined subjects. Moreover, the respondents in the present study were not very precise in their answers to this particular question, and the obtained results have to be treated as an estimate. It was demonstrated that the presence of erosion in the anterior segment was associated with the type of toothbrush and the hardness of the fibers. An increased severity of lesions was observed in subjects using electric brushes compared to manual ones. No statistically significant differences were observed regarding the preferred method of brushing, use of fluorides, or reported dental hypersensitivity.

In a study conducted by West et al., tooth sensitivity was strongly correlated with the occurrence of dental erosion [34]. In the present study, there was no such correlation. The reason for this variation could be the relatively low lesion severity, a younger age of assessed group/population, and small percentage of people declaring tooth sensitivity.

It has been demonstrated that the most common risk factors for the onset of dental erosion in a young adult population in Poland include gender, place of residence, preferred diet, hygienic habits and practices, and medical conditions such as asthma, eating disorders, and esophageal reflux.

Based on the current study, the risk factors for erosion among the Polish young adults’ population can not be fully determined. The analysis was conducted on the cohort of 18-year olds that is not a fully representative group. In addition, many factors such as lifestyle habits (swelling, drinking habits) and sport activities were not investigated in the study. Further studies conducted across the young adult group (18–35 years old) are necessary in order to conclude a frequency, intensity, and risk factors of the erosion in the study group.
